# Supplementary material for: Quantitative evaluation of muscle mass based on chest high-resolution CT and its prognostic value for tuberculosis: a retrospective study
Source: PeerJ. 2025 Mar 17;13:e19147. doi: 10.7717/peerj.19147 (PMC11925048; doi:10.7717/peerj.19147)
Supplement: Supplemental Information 4 [file peerj-13-19147-s004.docx]

Table S2. Variance Inflation Factor for the Multivariable Linear Regression Analysis of the Whole Lung Lesion Absorption Ratio

| **Variables** | **VIF** |
| --- | --- |
| T12 SMI | 1.368855 |
| T12 SMRA | 2.263896 |
| Age | 1.728194 |
| Gender (Female) | 1.784351 |
