# Supplementary material for: Quantitative evaluation of muscle mass based on chest high-resolution CT and its prognostic value for tuberculosis: a retrospective study
Source: PeerJ. 2025 Mar 17;13:e19147. doi: 10.7717/peerj.19147 (PMC11925048; doi:10.7717/peerj.19147)
Supplement: Supplemental Information 5 [file peerj-13-19147-s005.docx]

Table S3. Variance Inflation Factor for the Multivariable logistic Regression Analysis of Factors Associated with Poor Prognosis

| **Variables** | **VIF** |
| --- | --- |
| T12 SMI | 1.342871 |
| T12 SMRA | 1.949426 |
| Age | 1.61415 |
| Gender (Female) | 1.686984 |
