# Supplementary material for: Quantitative evaluation of muscle mass based on chest high-resolution CT and its prognostic value for tuberculosis: a retrospective study
Source: PeerJ. 2025 Mar 17;13:e19147. doi: 10.7717/peerj.19147 (PMC11925048; doi:10.7717/peerj.19147)
Supplement: Supplemental Information 6 — ROC, receiver operating characteristic; AUC, the area under ROC curve; CI, confidence interval. [file peerj-13-19147-s006.docx]

Table S4. Comparing the AUC under different ROC curves using Delong’s test

|  | **Difference between AUC (95% CI)** | **p-value** |
| --- | --- | --- |
| **T12 SMI vs BMI** | 0.061 (-0.010, 0.13) | 0.091 |
| **BMI vs T12 SMRA** | 0.098 (-0.024, 0.222) | 0.116 |
| **T12 SMI vs T12 SMRA** | 0.159 (0.065, 0.2544) | <0.001 |
| ROC = receiver operating characteristic; AUC: the area under ROC curve; CI = confidence interval. | | |
